# Pursuing Artful Movement Science in Music Performance: Single Subject Motor Analysis With Two Elite Pianists

**DOI:** 10.1177/00315125211003493

**Published:** 2021-03-27

**Authors:** Craig Turner, Peter Visentin, Deanna Oye, Scott Rathwell, Gongbing Shan

**Affiliations:** 1 Department of Kinesiology and Physical Education, University of Lethbridge, Lethbridge, Alberta, Canada; 2 Department of Music, University of Lethbridge, Lethbridge, Alberta, Canada

**Keywords:** piano performance, biomechanical modeling, compensatory motor behavior, anthropometry, trunk movement, musical context

## Abstract

Piano performance motor learning research requires more “artful” methodologies if it is to meaningfully address music performance as a corporeal art. To date, research has been sparse and it has typically constrained multiple performance variables in order to isolate specific phenomena. This approach has denied the fundamental ethos of music performance which, for elite performers, is an act of interpretation, not mere reproduction. Piano performances are intentionally manipulated for artistic expression. We documented motor movements in the complex task of performance of the first six measures of Chopin’s “Revolutionary” Etude by two anthropometrically different elite pianists. We then discussed their motor strategy selections as influenced by anthropometry and the composer’s musical directives. To quantify the joint angles of the trunk, shoulders, elbows, and wrists, we used a VICON 3 D motion capture system and biomechanical modeling. A Kistler force plate (1 N, Swiss) quantified center of gravity (COG) shifts. Changes in COG and trunk angles had considerable influence on the distal segments of the upper limbs. The shorter pianist used an anticipatory strategy, employing larger shifts in COG and trunk angles to produce dynamic stability as compensation for a smaller stature. Both pianists took advantage of low inertial left shoulder internal rotation and adduction to accommodate large leaps in the music. For the right arm, motor strategizing was confounded by rests in the music. These two cases illustrated, in principle, that expert pianists’ individualized motor behaviors can be explained as compensatory efforts to accommodate both musical goals and anthropometric constraints. Motor learning among piano students can benefit from systematic attention to motor strategies that consider both of these factors.

## Introduction

Instrumental music performance ranks among the most complex of learned human behaviors (Visentin et al., 2015). For example, a professional pianist performing the 11th variation of Franz Liszt’s 6th Paganini-Etude must play up to 1800 notes per minute for some sections of the music (Münte et al., 2002). Performing in a Wagner opera can take 4½ hours (Wagner, 1868). Given the physical intensity, low tolerance for errors, and high endurance requirements of music performance, it has been categorized by many as an athletic endeavour (Dick et al., 2013; Quarrier, 1993). However, unlike athletes, musicians typically receive little or no education regarding the most effective ways to prepare their bodies and minds for the rigors of performance (Wijsman & Ackermann, 2019). Rather, instruction in the mechanics of playing an instrument is typically based primarily on a teacher’s experience. The quantitative literature in human movement science pertaining to music pedagogy and motor learning is only now beginning to emerge (D’Amato et al., 2020; Furuya & Altenmüller, 2013; Visentin et al., 2015).

The dearth of human movement research in music instruction may be explained in part by cultural norms in the music discipline. Even today, most western classical music pedagogy relies upon centuries-old, tradition-based, master-pupil teaching strategies that are, for the most part, only nominally systematic (Purser, 2005; Norton et al., 2015; Visentin et al., 2015). Although this model of pedagogy has offered benefits of individualized training, so strong a reliance on teachers’ personal perceptions of their own experiences has pedagogical limitations. Another reason for scarce motor learning research in music training is likely an artifact of disciplinary and cultural boundaries in human movement science. With its origins in sports analysis and daily living activities, human movement science methods and analytic techniques are best suited for examining movement repeatability. Yet, at its most artistic, music performance, like the highest levels of sports performance, is an act of interpretation and perhaps even improvisation, not one of reproduction or utility (Cook, 2014; Palmer, 1997; Shan & Visentin, 2010). Outcomes in music performance are intentionally manipulated toward artistic expression. For musicians, a failure to consider musical context when analyzing underlying motor behaviors renders research pointless for real-world applications. For human movement scientists, artistic manipulation of context can be a nearly insurmountable confounding factor in experimental designs and data analysis (Shan et al., 2007). This makes applying human movement research methodologies to motor learning when playing a musical instrument very challenging. If music performance is the central object of study, music motor behavior research must become more “artful” in its analytic motivation and methods so that research design informs artistry rather than merely describing performance gestures.

Despite seeming incompatibilities between music performance instruction and human movement science to date, there is a small but growing body of research that has applied human movement science methods to music performance (Baadjou et al., 2017; Ferrario et al., 2007; Hopper et al., 2017; Rickert et al., 2013; Shan & Visentin, 2010; Visentin et al., 2008). Because music performance is a task with high perceptual motor demands, a musician’s gross *and* fine motor control are visibly co- and inter-dependent, notwithstanding intentional artistic interpretive variability (Shan et al., 2013). Thus, elite musicians must learn a variety of fundamental motor movements and strategies, and practice manipulating them, in order to render performances that are novel while still falling within expectations of musico-cultural traditions. Motor learning research has the potential to accelerate motor learning by informing traditional experience-based pedagogical methods with scientific analysis and objective reasoning, so long as science remains sensitive to musical performance demands. Although the existing literature has shown, in principle, that human movement science methods have analytic utility for describing elements of music performance, the next step in applying human movement science to music instruction must be to demonstrate goals of artistic flexibility are not encumbered by limitations of experimental research design.

For improving piano performance, most biomechanical and motor behavior research to date has employed protocols that emphasize reductionistic keystroke exercises (Furuya & Kinoshita, 2008; Degrave et al., 2020; Oku & Furuya, 2017; Verdugo et al., 2020). Some have used scales, which are mechanical exercises designed to develop a pianist’s technique (Ferrario et al., 2007; van Vugt et al., 2012, 2014). A smaller number of studies have examined piano performance in the context of musical excerpts. Most of these have controlled performance variability by instructing performers in “how” to perform the music so that non-expressive and expressive performances can be distinguished (Castellano et al., 2008; Thompson & Luck, 2012; Massie-Laberge, Cossette, et al., 2018; Massie-Laberge, Wanderley, et al., 2018). The methodologies of these studies have illuminated some of the mechanical demands of piano performance, but they have not addressed artistic demands because they have restricted or modified performers’ subconscious musical intentions and, concomitantly, the motor behaviors associated with them. Although researchers highly value controls over variability beyond factors of primary interest, the reality of concert performance is that the musical context drives performance variables; individual musical intention necessarily influences the selection of specific motor behaviors.

From a biomechanical perspective, anthropometry is important when learning a skill. Interestingly, with the exception of research on hand span and ergonomically modified keyboards (Booker & Boyle, 2011; Boyle et al., 2015; Chi et al., 2020; Deahl & Wristen, 2017; Farias et al., 2002; Lai et al., 2015; Wagner, 1988; Wristen et al., 2006; Yoshimura & Chesky, 2009), anthropometry has been overlooked in existing biomechanics research on piano performance and music pedagogy. Factors suggesting a need for more attention to anthropometry include these: (a) the keyboard is immobile and of fixed dimensions, (b) pianists must play notes according to directives in the musical score, and (c) anthropometry is largely a fixed variable for each pianist who must individualize positioning and repositioning the body to facilitate how fingers address the keyboard during performance. Within this individualization, some generalizable trends may exist.

Developing motor learning strategies that are appropriate for an individual’s anthropometry will ultimately allow pianists to optimize their performance outcomes (i.e., achieve autonomous motor learning sooner) (Fitts & Posner, 1967). Given the significance of gross motor movement on fine motor execution and that pianists’ gross motor movements have been understudied, we aimed, in this study, to address anthropometry and musical context for pianists’ gross motor behaviors. One of the few studies in this realm analyzed trunk motion in pianists (Verdugo et al., 2020), another analyzed hip kinetics (Massie-Laberge, Wanderley, et al., 2018), and several recognized core balance/center of gravity (COG) as an important factor in piano performance (Koga & Nogami, 2012; Wristen, 2000; Zhang, 2020). Drawing on this preceding research, we examined two elite pianists’ motor behaviors during a complex musical performance so as to quantify pianists’ joint activity in the trunk, shoulders, elbows, and wrists and their dynamic balance shifts in COG. In our discussion of observed results, we then considered the individual anthropometric and musical drivers that may have motivated their motor strategies.

## Method

### Participants

In this multiple single subject analysis, we recruited two anthropometrically different, right-handed, expert pianists as study participants (one female and one male). Participant 1 (A) was 1.6 meters tall and Participant 2 (B) was 1.9 meters tall. Neither participant was hypermobile (Beighton Hypermobility protocol). Both pianists held doctoral degrees in piano performance and were informed of the data collection procedures and research protocol prior to providing their written informed consent (University of Lethbridge Human Subject Research Committee approval #2018-098).

### Materials

**Figure 1.**
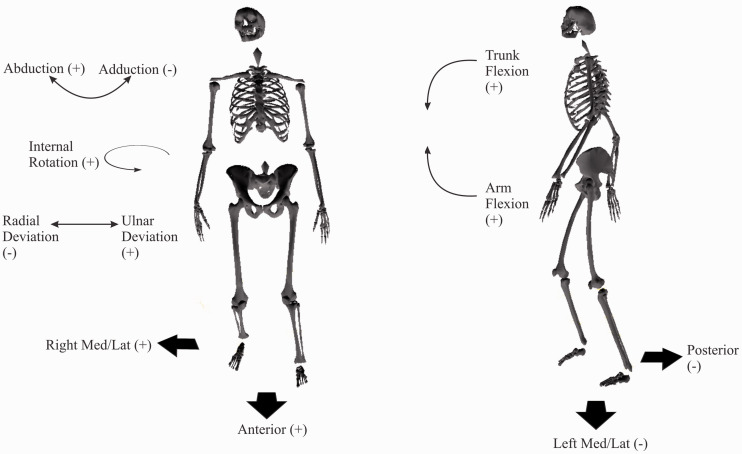
Upper Arm, Trunk, and COG Neutral Positions According to the Standard Anatomical Position. Note: Trunk flexion, shoulder flexion, abduction, internal rotation, arm flexion, wrist flexion, and ulnar deviation are positive angles. COG excursions that are anterior and to the right (upper end of the keyboard) are positive.

A ten-camera 3 D motion capture system (VICON MX40, Oxford, England) was used. Thirty-nine reflective markers were placed on the participants in accordance with a 15-segment full-body biomechanical model. From positional data, this model permitted joint angle and range of motion (ROM) quantification of the pelvis (trunk), shoulder, elbow, and wrist (Figure 1). Data was recorded at 200 frames/s with a calibration error of <0.6 mm. Marker placements were: four on the head, nine on the trunk (sternal end of the clavicle, xiphoid process, C7, T10, right scapula, and the right and left anterior superior iliac and posterior superior iliac), 14 on the upper extremities (the left and right acromion, lateral side of the humerus, lateral epicondyle, lateral side of the forearm, radial styloid process, ulnar styloid process, and 3rd metacarpal), and 12 on the lower extremities (the left and right lateral side of the thigh, lateral tibial condyle, lateral side of the shank, lateral malleolus, calcaneus, and distal end of the hallux). Participants wore a specialized garment that permitted secure marker placement without impeding movement. Additionally, 88 markers were placed on both white and black piano keys to identify keystroke accuracy.

**Figure 2.**
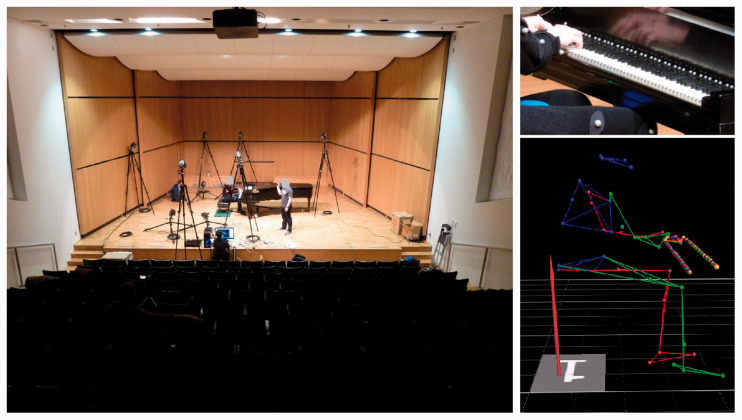
The Experimental Set-up (Left), With Markers Placed on the Participant and Keyboard (Top Right), and a Sample Frame From the Computer-Generated Model (Bottom Right).

We placed a Kistler force plate (1 N, Swiss; 60 cm × 40 cm) under the piano bench to measure the participants’ anterior/posterior (ant/post) and medial/lateral (med/lat) COG shifts. Force plate data employed the center of the plate as the origin for COG measurements, and was synchronized with motion capture data. Figure 2 shows the experimental set-up and illustrates a sample frame from the computer-generated participant and keyboard models.

### Musical Excerpt

**Figure 3.**
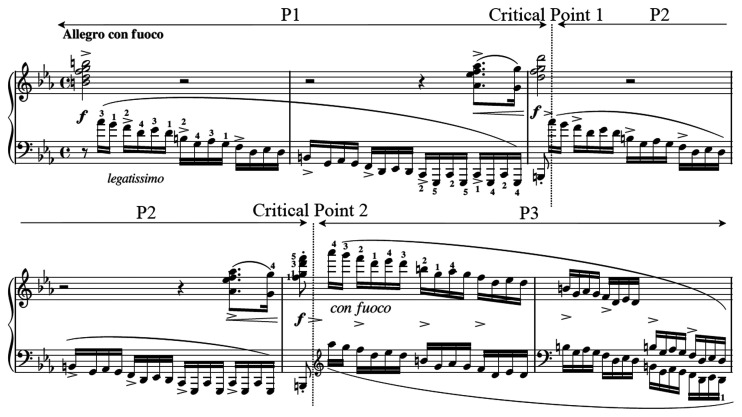
The First Six Measures of Chopin’s Op. 10, No. 12 (“Revolutionary Etude”) Performed by the Participants. Note: The vertical lines (critical points) indicate the moment when the pianist must shift across the keyboard. P1, P2, and P3 are labelled accordingly.

Participants performed the first six measures of Chopin’s “Revolutionary” Etude Op. 10, No. 12 (Figure 3). Participants were given the music two weeks prior to data collection. This excerpt was chosen because it is extremely difficult, and the musical context demanded a variety of motor skills. This also naturally divides into three parts (P1, P2, and P3), demarked by two critical points where discontinuous leaps from the low to high registers of the piano occur (Figure 3, dotted lines). For the left hand (bottom notes on each musical stave), motor demands are continuous throughout all segments. For the right hand (top notes on each stave), motor demands are tripartite in P1 and P2, with playing a chord two beats in length, resting (periods where the hand is not playing any notes) five beats in length, and a “pickup” or leading gesture into the next segment, one beat in length. In P3, the right hand mimics the general pattern of the left hand. These motor skills, both symmetric and asymmetric, required highly developed motor coordination between both limbs, something that is achieved through long-term training (Kilincer et al., 2019).

Unlike many other activities involving keystroke manipulation (such as typing at a computer keyboard), playing legato (smoothly) at the piano requires precise coordination of the downward movement of one finger (to sound a note) with the upward movement of another (to stop the note that is already sounding). Chopin’s musical directives required an allegro con fuoco (fast with fire) tempo (playing speed), legatissimo (the smoothest possible) articulation, a forte (loud) volume, accents (>) on specified notes, localized crescendi (increases in volume), and chordal structures requiring simultaneous use of four or five fingers of the right hand.

In the current study, performance tempo was controlled at 135 beats per minute (bpm), using a metronome. Since four notes per beat are required in this composition, resultant playing speed was 540 notes/min or roughly 9 notes/s (N/s). At this tempo, the musical excerpt was fast enough (110 ms per keystroke during the 16th notes) that performers could not possibly execute each note as an individual gesture. According to Rottondi et al. (2016), variability in musical timing below 20 ms is perceived by listeners as highly coordinated, and according to Kazennikov and Wiesendanger (2009), differences between 60 and 100 ms are perceived as errors in timing. Considering these constraints, the musical excerpt of the current protocol left performers virtually no room for error.

### Procedure

Participants performed on a 9-foot New York Steinway grand piano in a concert hall setting. Unlike artificial keyboards/digital pianos where significantly less force is required to depress a key and tone quality may be compromised due to electronic sound production (Meinke, 1995), a Steinway grand piano is generally accepted as an ideal concert performance instrument for professional pianists. Subjects were permitted to warm-up and adjust bench height/position according to personal preference. Anthropometric measurements (body height, body mass, leg length, ankle and knee width, shoulder offset, elbow and wrist width, and hand thickness) were documented prior to testing for the purposes of biomechanical modeling.

### Data Processing and Analysis

We used a 15-segment biomechanical model to process raw kinematic data with VICON Nexus Software. The model employed a rigid-body system with multiple segments: head, upper trunk, lower trunk, upper arms, forearms, hands, thighs, shanks, and feet. Using established anthropometric norms (Shan & Bohn, 2003; Winter, 1990), we calculated inertial characteristics of segments. We analyzed data with Microsoft Excel software to determine center of gravity (COG), joint angles and joint range of motion (ROM) for the pelvis (trunk), shoulders, elbows, and wrists.

## Results

Table 1 displays the trunk, shoulder, elbow, and wrist ROMs, as well as the COG excursion for both participants. Clearly, each pianist (A and B) utilized a different motor behavior strategy to perform the excerpt. For nine of the fourteen measured joint angles, A used greater ROM than B. For seven of the fourteen joint angles, the difference was notable (greater than 5°, with right shoulder flex/ext differing 28.7°). For B, only two joint angles showed notably larger ROM than A (right shoulder abduction/adduction (ab/add) and left wrist flexion/extension (flex/ext)). Five joint angle ROMs were very similar between participants (left shoulder flex/ext and rotation, right shoulder rotation, left elbow flex/ext, and left wrist rad/uln). COG excursion was much larger for A in the medial/lateral (med/lat) plane while it was larger for B in the anterior/posterior plane (ant/post) (295.0 mm vs 209.6 mm, and 51.1 mm vs 43.0 mm, respectively).

**Table 1.** Upper Body Joint Angle ROM and COG Excursion ROM for Both Pianists.

	Participant A	Participant B
Trunk (°)		
Ant/Post	**16.2**	10.0
Med/Lat	**50.9**	36.7
Left shoulder (°)		
Flex/ext	30.3	30.4
Ab/Add	**44.7**	36.1
Rotation	46.2	45.2
Right shoulder (°)		
Flex/ext	**49.6**	20.9
Ab/Add	19.0	**27.0**
Rotation	43.4	46.1
Left elbow (°)		
Flex/ext	36.6	33.7
Right elbow (°)		
Flex/ext	**42.5**	26.9
Left wrist (°)		
Flex/ext	29.6	**39.9**
Rad/uln	17.0	21.3
Right wrist (°)		
Flex/ext	**50.5**	30.8
Rad/uln	**21.8**	13.5
COG (mm)		
Ant/Post	43.0	**51.1**
Med/Lat	**295.0**	209.6

*Note*: Numbers in bold indicate the subject with the larger ROM in cases where large ROM differences exist.

**Figure 4.**
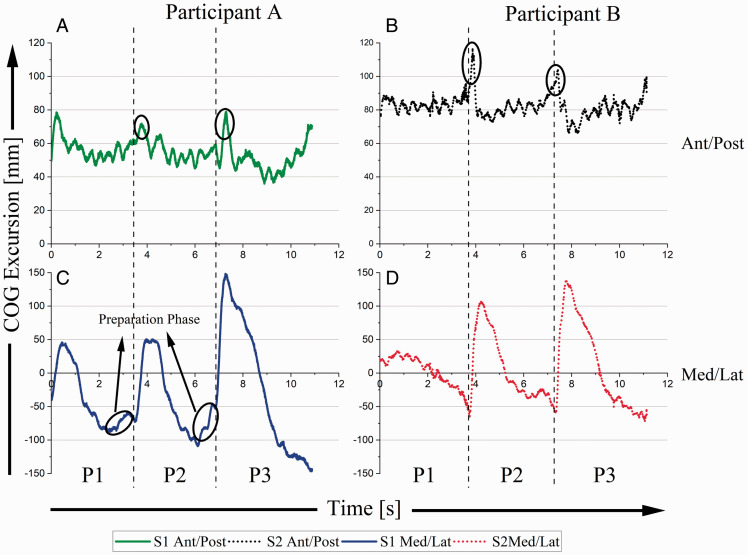
Anterior/Posterior and Medial/Lateral COG Positions as a Function of Time for Both Pianists. Note: An increase in COG excursion represents shifts that are anterior and to the right. In 4A and 4B, circled peaks signify sudden anterior shifts (movement toward the keyboard) corresponding to discontinuous leaps in the music. In 4C, circled areas signify preparation phases.

Figure 4 shows COG ant/post and med/lat excursions (measured from the force plate origin) for each participant. A positioned the bench 10.8 cm closer to the keyboard and 1.1 cm further to the right than B’s bench position. A’s bench height was approximately 2.8 cm higher than B’s bench height. Starting body positions for the performers were also different. A’s starting COG position relative to middle C on the keyboard was 0.7 cm closer, 2.3 cm further to the left, and 3.1 cm lower than B’s starting COG position (A’s coordinates: 20.8 cm, 4.9 cm, −0.2 cm; B’s coordinates: 21.5 cm, 7.2 cm, 2.9 cm).

For both participants, changes in COG ant/post were small throughout the musical excerpt, 43.0 mm (A) and 51.1 mm (B) (Figure 4A and B). However, larger movements occurred at the critical points (Figure 4, vertical dotted lines) where discontinuous left-hand leaps were required by the music. This showed both performers to be shifting balance toward the keyboard (Figure 4A and B, circled peaks). With respect to COG med/lat movement (Figure 4C and D), there were large and notable shifts to the right at critical points. COG med/lat excursions (Figure 4C and D, trough to peak) increased for each consecutive section of the music (P1, P2, and P3). COG med/lat excursions for A (Figure 4C, trough to peak) were 86 mm, 136 mm, and 256 mm, respectively. For B (Figure 4D, trough to peak), they were 24 mm, 174 mm, and 199 mm. Notably, at P1 and P2, where the musical demands were nearly identical, A’s peak med/lat COG shifts were nearly identical, whereas those for B were not. Both participants utilized a larger med/lat COG shift for the last critical point.

**Figure 5.**
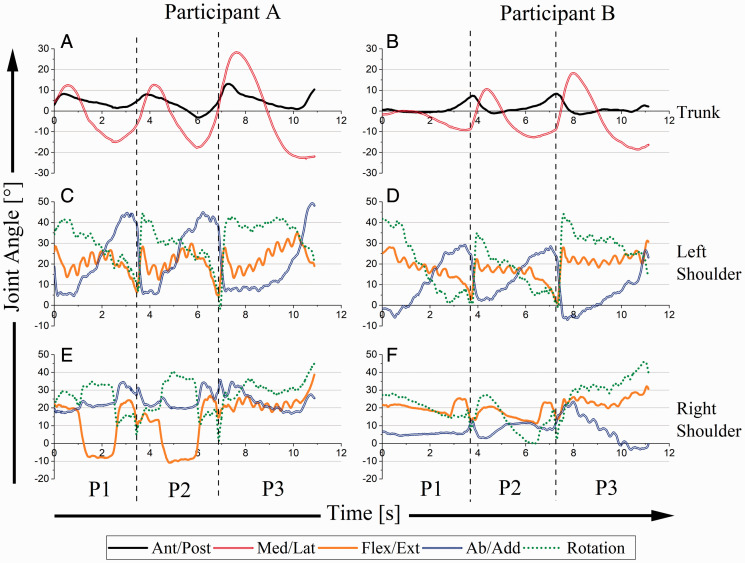
Trunk and Shoulder Joint Angles as a Function of Time for A and B. Note: Increased angles signify flexion (trunk and shoulders), side flexion to the right (trunk), abduction, and internal rotation (shoulders).

Figure 5 shows trunk and shoulder joint angles. For both performers, trunk angle graphs reinforce COG excursion findings; trunk movement increased for each consecutive part of the music (P1, P2, and P3). However, unlike COG findings where shifts of balance occurred at critical points and in a discontinuous manner, changes in trunk angle were gradual, controlled, and continuous.

For the left shoulder, participants employed similar motor behavior strategies. Ab/add and rotation had complementary functionality during P1, P2, and P3; as one increased (or decreased), the other decreased (or increased). Flex/ext cycles for A were wave-like, with peak flexion occurring in the middles of P1 and P2. For B, flex/ext showed a steady progression from greater to lesser flexion in each of P1 and P2. In P3, flex/ext increased steadily for A and was stable for B. At critical points, both participants utilized rapid left shoulder internal rotation and adduction.

Right shoulder motor behavior strategies were markedly different between participants. For A, right shoulder rotation increased (showing internal rotation) and shoulder flex/ext decreased (showing extension) during P1 and P2 (green and orange lines, Figure 5D). As well, shoulder ab/add increased slightly (showing abduction) at critical points. During P3, motor behavior in all three joints stabilized in narrow ranges. For B, right shoulder rotation decreased (external rotation) and ab/add increased (abduction), during P1 and P2 while there was a complementary exchange of roles between ab/add and rotation in P3 (green and blue lines, Figure 5C). Flex/ext usage appeared to be unperturbed throughout P1, P2 and P3.

**Figure 6.**
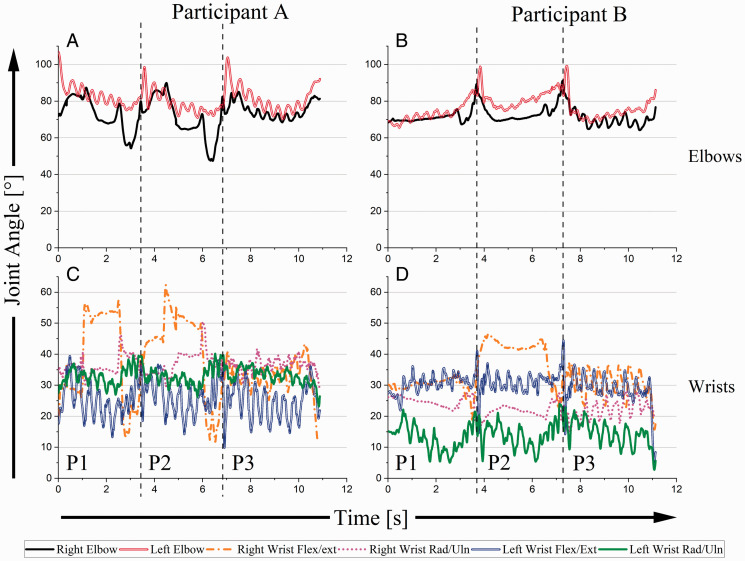
Elbow and Wrist Joint Angles as a Function of Time for A and B. Note: Increased elbow angles represent flexion while increased wrist angles represent flexion and ulnar deviation.

Figure 6 shows joint angles for the elbows and wrists. For each performer, localized oscillations in the elbows and wrists were larger than those observed in the shoulders. For both performers, left elbow angles spiked suddenly (showing flexion) at critical points (red lines, Figure 6A and B). However, during each of P1, P2, and P3, participants’ motor behaviors were opposite; A’s left elbow angle decreased (showing extension) during each of these segments, while B’s increased (showing flexion). Right elbow movement for A was much larger than for B (black lines, Figure 6A and B). For both participants, there was an anticipatory elbow movement leading to critical points; this strategy was more pronounced for A than for B. Right and left elbow joint movement was independently asymmetrical for A in P1 and P2. In P3, elbow movement became more symmetrical. For B, right and left elbow movement was more symmetrical throughout P1, P2, and P3. In the wrists, A used a right wrist flex/ext strategy throughout P1 and P2 (orange line, Figure 6C). In P3, A’s wrist joint angles were stabilized. For B, flex/ext was consistently greater than rad/uln during P1, P2, and P3 (Figure 6D).

## Discussion

In the present study, we examined two expert pianists’ gross motor behaviors while performing the complex opening of Chopin’s Revolutionary Etude, and we analyzed the activity of the trunk, the joints of the shoulders, elbows, and wrists, and we quantified dynamic balance shifts in center of gravity (COG). We postulate that our use of elite pianists and a composition from the virtuosic literature led to performances that were influenced more by musico-cultural traditions and expectations than by experimental conditions. Performers’ individual approaches were not constrained except by tempo and were apt to reflect their individualized artistic expression. Thus, the composer’s musical directives, the performer’s anthropometry, and the performers’ motor strategies were all manifested in these data.

ROM data provided a general overview of each pianist’s motor behavior strategy. Given the detailed musical directives in the score of the Revolutionary Etude, it might seem that motor behavior would be limited to a single possible strategy. Clearly, this was not the case, as each pianist used an individualized motor behavior strategy. To understand *how* each pianist employed gross motor behavior strategies in the service of a musical outcome, we analyzed: COG position, trunk movement, and shoulder, elbow, and wrist joint angles.

### COG and Trunk

A adjusted the bench to be higher and closer to the keyboard compared to B. Starting COG positions for both pianists were indicative of their seating location; A was 0.7 cm anterior, 2.3 cm left, and 3.1 cm lower, compared to B’s starting position. To some extent, bench position/height and starting COG positions may be explained by anthropometry. B was significantly taller than A, and positioning of the bench had to be further away from the piano for B’s legs and arms to be in a comfortable orientation to the keyboard. However, anthropometry does not explain some of the motor control differences observed during the excerpt performance.

Within the music, P1 and P2 are nearly identical. A treated P1 and P2 with a consistent mechanical process, with shifts in both COG and trunk angles during P1 and P2 showing nearly identical contours (Figure 4A and C; Figure 5A). On the other hand, for B, COG and trunk angles showed markedly different mechanical processes during P1 and P2 (Figure 4B and D; Figure 5B), with B’s COG and trunk angles looking more like those of A during P2. Clearly, the two participants used different starting approaches in their performances. A was more anticipatory in preparing the start than B, but by the time P2 occurred B had adapted his motor strategy to the demands of the composition. To some extent, anthropometry may have played a role. Since this composition required large lateral movements of the left arm across the body, A’s shorter stature may have necessitated her anticipatory movement strategy. For B, a greater reach, because of his taller stature, might have made this less imperative. But, clearly by P2, B had modified his strategy to one that was more similar to that of A. Perhaps the difference between “viable” and “optimal” motor strategies explains this change.

At critical points, large physical movements needed to occur. At the first critical point, the left hand was required to move medial/laterally a distance of 48 cm, and at the second critical point it needed to move 65 cm. These movements occurred in less than 0.22 seconds. Shifts in COG can provide insight into motor strategies in this regard; differences between participants can be explained by both musical demands and anthropometry.

Both participants’ COG shifted anteriorly, towards the keyboard, at critical points. Right and left hands were one octave apart, and bringing COG closer to the keyboard provided a means of leveraging body weight into the arms to assist the creation of a forte (loud) sound (spikes in graph contours of Figure 4A and B). Increases in med/lat COG excursions (trough to peak) at the second critical point may be partly explained by musical demands; the left hand needed to move one octave further on the keyboard. Notwithstanding musical constraints, anthropometry may help rationalize motor strategy differences between participants. A (the shorter pianist) utilized a preparatory strategy in anticipation of the large leaps at critical points (Figure 4C). There was no evidence of this for B, whose preparatory strategy did not require this adjustment, as he could reach further across the keyboard. Anthropometric differences may also explain the med/lat ROM disparity between participants (Table 1; A = 295 mm, B = 210 mm). A’s larger COG movement may have been a compensation for a shorter reach.

Regardless of anthropometry, the trunk orients body position for all motor behaviors (Magill & Anderson, 2017). During piano performance, changes in trunk angle (COG position) has a concomitant effect on arm movement (Verdugo et al., 2020). Manipulation of trunk and arm angles determines hand-keyboard orientation. Given that piano performance requires large changes in these variables as well as symmetrical and asymmetrical changes among these variables, trunk stability must be dynamic. This explains why, in the current study, participants’ trunk angles changed in a gradual and controlled manner. The differences in control between participants may be explained by anthropometry. Particularly, for A, dynamic trunk stability employed a preparatory strategy. This preparatory strategy is biomechanically efficient because it takes less effort to move proximal body segments than distal ones (simply, there is less torque), and a preparatory strategy helps the performer achieve an earlier upper limb skeletal alignment, facilitating fine control of the fingers. Dynamic stability of the trunk influenced shoulder, elbow, and wrist motor behavior strategies for both pianists. From a musician’s standpoint, whether realized or not, gross motor movements must either be a response to (occurring after) or a preparation for (occurring before) musical demands. Strategy selection influences interpretive outcome.

### Shoulder

For both participants, left shoulder ab/add and rotation were complementary; left arm abduction and external rotation were used in the movement strategy during P1 and P2. In general, there is no utility in moving the left arm in the flex/ext plane because the keyboard and bench height are fixed. A scenario in which significant manipulation of shoulder flex/ext might occur would be when the arm needs to accommodate trunk position (i.e., arm must move in front of the trunk, resulting in shoulder flexion). A’s use of a preparatory trunk movement strategy can be observed in flex/ext of the shoulder (Figure 5C, orange line). Whereas her shorter stature generally required flex/ext to increase (flex) for the descending left-hand musical patterns, anticipatory trunk movement permitted her to reduce flexion at the ends of P1 and P2. For B, left shoulder flex/ext decreased throughout each P1 and P2 because there was no preparatory trunk movement; flex/ext increased suddenly in coordination with the large left-hand leaps.

For the left shoulder both participants utilized rapid internal rotation and adduction to accommodate large left-hand leaps at critical points. This strategy takes advantage of the low inertial properties of shoulder rotation, enabling fast and easy arm movement across the keyboard without negatively affecting “smoothness” of arm control in the distal segments. We suspect that both participants utilized the same strategy because of timing constraints. Since movement at the critical points needed to occur in less than 110 ms, using internal rotation and adduction made the passage possible. Any strategy that involved larger movement of distal segments would have taken more physical effort (given a non-infinite availability of physical force, more effort means more time).

For the right shoulder, each pianist used different motor strategies. This was a product of the manner in which they chose to utilize musical rests (when no notes are being played) during P1 and P2. Right hand behavior during P1 and P2 can be divided into three distinctive sections: (a) musical rests, (b) a “pickup”, or gesture leading into, (c) a chord. A used shoulder extension and internal rotation during the musical rests (“active rest”) as a mechanism to prepare for the leading gesture, whereas B used external rotation and abduction. A used the rests as opportunities to “relax” the right limb, choreographing its re-entry into the musical context just before it was needed. For A the right and left limbs operated independently. B maintained playing preparedness in the right limb throughout the rests. In this manner, the right and left limbs operated more dependently. Both of these choreographic strategies have utility. Certainly, for short periods of rest, an active hand choreography should facilitate musical fluency. For longer periods of rest, especially for a composition of some significant length, utilizing musical rests as opportunities to relax muscle groups may delay fatigue and reduce its concomitant and negative impact on musical precision and outcomes. The excerpt used in the current protocol was short, and the right limb rests lent themselves to either option. Of course, choice of strategy in this regard necessitates differences in COG shifts.

For A, the act of putting her right hand on her right thigh during musical rests was a means to rest the right shoulder and arm muscles. For B, choreographing the right arm in and out of the musical gesture proved to be a joint activity minimization strategy for both limbs. By resting the right limb, temporarily removing it from the musical gesture, all effort was focused on left-limb execution, permitting small joint movements with a concomitantly larger COG shift in the trunk. When only considering the moments where the right limb was active (not resting on the thigh), all joint angles exhibited narrow ROM, showing considerable motor efficiency. For B, maintaining right arm activity throughout the rests allowed him to reduce right arm joint movement in a different manner. For B, there was no need for a timing choreography to reintroduce the right arm into the musical gesture. His larger reach permitted this as well as smaller COG movement.

### Elbow

At critical points, both participants flexed (increased joint angle) the left elbow to accommodate for large leaps in the music. During all P1, P2, and P3, A used extension of the left elbow (decreased joint angle) to guide the left hand as it descended the keyboard. Greater COG ROM facilitated this. B utilized elbow flexion as his arm “reached” down the keyboard. Greater reach permitted smaller COG ROM.

Right elbow movement was different between participants because of the manners in which they used musical rests. For A, the right elbow was flexed during the rest period and extended during the “pickup” gesture in preparation for the chords. For B, right elbow angles mimicked those of the left elbow. This strategy reduced the complexity of limb movement in a passage of music that demanded asymmetrical arm movements.

### Wrist

Dynamic stability is observed through the oscillations in wrist joint angles – more distal joints (i.e. the wrists) exhibited greater oscillatory patterns compared to more proximal structures (i.e. the shoulders). For every accent in the excerpt there was an oscillation in the wrist to generate a louder/stressed sound. In terms of motor behavior, the accents “chunked” the fast notes into groups of four.

A used right wrist flexion to prepare right-hand chords at each critical point during the musical rest section. During P3, wrist joint angles were stabilized because the trunk was positioned between both hands, resulting in no need to excessively flex or deviate the wrist. For B, a larger hand size may have meant that flex/ext had greater utility than rad/uln because of increased “reach”.

### Limitations and Future Directions

The current study suggests that there are many successful strategies available to pianists to accomplish any given performance outcome. It may be that some strategies are more useful to some pianists, given anthropometric variability. The current study provides a framework for future research intending to analyze and train motor behaviors during piano performance. Ultimately, with a large enough body of evidence, such work can demystify complex motor behavior and strategizing during pedagogy and performance. Since this study analyzed the movements of only two elite pianists, it can only be considered a proof of principle, providing a starting point for future research that might possibly include the examination of additional anthropometric measures (e.g., hand span and finger lengths). Further, the current analysis only involved a small portion of a single composition. Finally, we made no attempt in this study to determine optimal performance movements or optimal training strategies for such complex activities. We assumed that these elite performers would provide a model against which further pedagogical research might compare the performances of students with similar anthropometry.

## Conclusion

In the current case comparison, both pianists displayed compensatory movements suitable for their own anthropometry and interpretations of musical demands. For both performers, shifts in COG and trunk position had considerable influence on the distal segments of the upper limbs. These shifts were used to enable rapid lateral hand movement. The shorter pianist used larger shifts in COG and trunk position than the taller one. This enabled her to foster a dynamic stability, effectively compensating for a smaller stature. This performer’s motor strategization was remarkably consistent throughout the excerpt, and anticipatory of the discontinuous melodic demands at critical points in the music (points where abrupt gross movement was required). Notably, A, used a COG shift even prior to her playing the first note of the composition, emphasizing that this strategy was preparatory rather than reactive. The effect of the above strategy was augmented by left shoulder movement, where rapid internal rotation and adduction was used to minimize the effort of playing the large left-hand leaps. This strategy takes advantage of the low inertial properties of shoulder rotation, which enables fast and easy arm movement across the keyboard. For the right arm, motor strategization was confounded by the presence of rests in the music; two performative possibilities existed: (a) to use the rest as an opportunity to temporarily relax muscle groups in the right arm, or (b) to maintain a directed right arm choreography throughout the rest. The two pianists of the current study chose different strategies and, correspondingly, motor control of the right-arm joints was very different. A used the opportunity to relax the arm, while B maintained a directed tension throughout the rests. No attempt was made to evaluate whether one of these strategies was “better” than the other however, in longer performances, the first strategy might better assist in fatigue management. With regard to the left and right wrists, the performer with the smaller hand size (A) used more rad/uln deviation while the performer with the larger hand size (B) used more flex/ext.

These results, as an initial investigation, might suggest that the personal ‘style’ and individual creativity of a performer can be derived from their development of a variety of motor behaviors that are compensatory in nature; accommodating body size and shape and motivated by outcomes that show individualized respect for the musical context. Thus, incorporating scientifically based motor learning strategies into complex piano pedagogy should help accelerate cognitive and perceptual motor skill acquisition and expand the range of motor behaviors available for student musicians seeking to manipulate motor movements in the service of artistic interpretation. Introducing this approach early in pedagogy may help learners avoid the acquisition of unnecessary muscular tensions and idiosyncratically inefficient motor behaviors.

We propose that elite pianists’ personalized motor behaviors are compensatory in nature; they adapt to affect the musical desires of the performer and are partially constrained by anthropometry. From a practical standpoint, analyses of motor behaviors will be most meaningful if they are sensitive to both a performer’s musical intentionality and his/her potential for motor strategization, developed over decades of practice. In the athletic endeavor that *is* music performance, augmenting knowledge of motor strategies has the potential to positively influence music teaching and learning and expand movement science methodologies, broadening the scope of both music and movement science disciplines.
